# Improving outcomes with anti-BCMA bispecific antibodies with attention to infection

**DOI:** 10.1038/s41408-024-01091-x

**Published:** 2024-07-08

**Authors:** Andrew J. Yee

**Affiliations:** 1 Massachusetts General Hospital Cancer Center, Boston, MA USA; 2 Harvard Medical School, Boston, MA USA

**Keywords:** Myeloma, Translational research

Therapies targeting BCMA are transforming the care of multiple myeloma, with unprecedented depth and durability of responses in patients with heavily treated disease. The field is moving rapidly, especially with the recent approval of anti-BCMA CAR T-cells in as early as one prior line of therapy with ciltacabtagene autoleucel (cilta-cel) and two prior lines of therapy with idecabtagene vicleucel. While patients are benefiting from the efficacy of these treatments, there is also growing data on the increased risk of infections with these therapies. In the initial trials of teclistamab (MajesTEC-1) and elranatamab (MagnetisMM-3), infections (all grade; grade 3–4; grade 5) were common with teclistamab (80%; 55.2%; 12.7%) and elranatamab (69.9%; 39.8%; 6.5%), respectively [1–3]. By comparison, infections were lower in the CARTITUDE-1 trial of anti-BCMA CAR T-cells cilta-cel (58%; 20%; 3%) [4, 5]. In the bispecific antibody trials, hypogammaglobulinemia (IgG <400 mg/dL) was also frequent with teclistamab (70.9%) and elranatamab (75.5%) [2, 3]. Moreover, opportunistic infections with *Pneumocystis jirovecii* and reactivation or infection with cytomegalovirus virus were seen on these trials.

The above findings are particularly relevant to multiple myeloma, which is already characterized by immune dysfunction and susceptibility to infection [6]. In a population study of 9253 patients, there was a 7-fold and 10-fold increased risk for bacterial and viral infections respectively, and 22.4% of deaths at one year were due to infection [7]. Anti-BCMA treatment further magnifies this risk. BCMA, in addition to being highly expressed on multiple myeloma cells, is also highly expressed on normal plasma cells and on B cells that are upstream of plasma cells as well as some B cell progenitors [8–10]. This accounts for the hypogammaglobulinemia seen with anti-BCMA therapy. Of note GPRCD, one of the newest focal points in multiple myeloma and the target of the recently approved bispecific antibody talquetamab, is also highly expressed in multiple myeloma and plasma cells, but unlike BCMA, is not expressed at a significant level on other immune cells [11, 12]. Interestingly, while talquetamab is associated with a similar level of hypogammaglobulinemia as seen in the MonumentTAL-1 study (IgG <500 mg/dL, 405 μg dose, 87%; 800 μg dose, 71%), the risk of infection appears significantly lower: grade ≥3, 14.2%. This raises the possibility that the expression of BCMA on cells outside of plasma cells as well as signaling downstream of BCMA may be important in conferring increased risk of infection with anti-BCMA bispecific antibodies, whereas the function of GPRC5D is less well established.

The work by Nath et al. in this publication provides additional insight into infection risks when targeting BCMA. The authors retrospectively analyzed 256 patients treated at Memorial Sloan Kettering Cancer Center with anti-BCMA treatment on a clinical trial or commercially: CAR T-cells, 36%; bispecific antibodies, 21%, and antibody drug conjugate, 43%. They found that the risk of grade ≥3 infection was significantly higher with bispecific antibodies, 40%, compared with CAR T-cells, 26%, and this also includes deaths from infections (7% v. 0%). In contrast, the risk of severe infection with ADC was appreciably lower, 8%. Moreover, there were differences in the timing of infections, with the risk for severe infections being higher in the first 100 days after CAR T-cells compared to bispecific antibodies (79% v. 50%), but this pattern reverses after six months. The ongoing infection risk with bispecific antibodies likely reflects the effects or repetitive treatment v a one-time therapy with CAR T-cells. Moreover, even though the duration of hypogammaglobulinemia was similar in both groups, the authors found that that incidence of any infection and high grade infection was higher with the bispecific antibody group, suggesting that the ramifications of hypogammaglobulinemia with bispecific antibodies are worse than with CAR T-cell treatment.

With growing experience using anti-BCMA bispecific antibodies, there are several approaches for mitigating the risk of infection. Intravenous immunoglobulin effectively treats hypogammaglobulinemia and reduces risk of infection. Several retrospective studies illustrate this. An analysis of 52 patients receiving teclistamab at Amsterdam University Medical Center compared patients receiving IVIG as primary prophylaxis (for IgG <400 mg/dL) v. who were observed with IgG <400 mg/dL and receiving IVIG only at time of severe infection [10]. The authors found that the cumulative incidence of serious infections at six months was significantly lower in the prophylaxis cohort, 5.3% v. 54.8% (*p* < 0.001). Similarly, at Mount Sinai in New York, investigators evaluated 37 patients receiving a variety of anti-BCMA bispecific antibodies [13]. IVIG was associated with 90% fewer grade ≥3 infections. Based on these observations, consensus recommendations are to give IVIG as primary prophylaxis when IgG <400 mg/dL [14, 15]. (Of note, older guidelines before the introduction of anti-BCMA therapy only recommended immune globulin replacement for hypogammaglobulinemia in patients who also had severe and recurrent infections [16]).

Another strategy is to optimize the dosing interval for bispecific antibodies. The initial approval for teclistamab specifies weekly dosing. The MajesTEC-1 study allowed patients to switch to every other week dosing in patients who achieved a partial response 4 cycles (phase 1) or CR or better after six months (phase 2) [17]. Most patients who switched (68.7%) continued with responses two or more years from the time of first response. Notably, spacing out the dosing to every two weeks reduced the rate of infections. There were fewer grade ≥3 infections in those who switched compared to those who remained on weekly dosing (15.6% v. 33.3%). This suggests that there are important benefits with reduction in infection beyond convenience. Based on these findings, in February 2024, the FDA approved spacing out teclistamab to every two weeks in patients who have achieved and maintained a complete response or better for a minimum of six weeks. In clinical practice, the change in interval happens sooner and not only in patients with a complete response. Other anti-BCMA bispecific antibodies in development, e.g. alnuctamab and linvoseltamab, move from weekly to every other week dosing earlier, at around cycle 4, and ABBV-383 is scheduled less frequently from the beginning, with dosing at q3 or q4 weeks in the phase 1 trial. Moreover, fixed duration of therapy is also under evaluation given the excellent durability of responses observed. For example, the LimiTEC study is evaluating discontinuation of teclistamab in patients who achieve a VGPR after 6–9 months of therapy [18]. Indeed, in addition to allowing for recovery of humoral immunity, lengthening the dosing interval may also importantly help address T-cell exhaustion with chronic, repetitive therapy and allow for improvement in T-cell function and efficacy [19].

A significant factor to address when discussing infection is COVID-19, and it is helpful to put the timing of when these trials enrolled patients in context. In fact, a dominant driver of grade ≥3 infections in MajesTEC-1 was COVID-19. The trial enrolled patients between March 2020 and August 2021, during the height of the pandemic, and only 7.9% of patients had received a COVID-19 vaccine prior to starting teclistamab [3]. The relatively higher rate of infection in MajesTEC-1 likely reflects the effect of COVID-19 in a largely unexposed, unvaccinated study population, as a significant proportion (35 of 91 patients with grade 3–4 infections and 18 of 21 deaths) were due to COVID-19. Now, the hazard of COVID-19 infection has been significantly attenuated with nearly universal exposure and/or vaccination along with antiviral therapies such as nirmatrelvir/ritonavir and remdesivir. The improving COVID-19 landscape as well as application of supportive therapies perhaps accounts for the reduced incidence of serious infections observed in real world use of teclistamab. In two series—one from five US academic centers and the other from 18 centers in Germany—grade ≥3 infections were lower compared to the MajesTEC-1, 17.3% and 26.8%, respectively [20, 21].

Anti-BCMA bispecific antibodies are now an important tool for treating multiple myeloma, and they have key advantages compared with CAR T-cell therapy, such as availability off-the-shelf and ease of administration compared to the more complicated logistics of the latter. Use of anti-BCMA bispecific antibodies is only expected to broaden as their use is being evaluated in earlier lines of therapy and in newly diagnosed patients, and in combinations with other agents. As is true with myeloma therapy in general, optimizations with respect to schedule and supportive care will evolve as we gain more experience. In summary, current recommendations include [14, 15]: maintain IgG >400 mg/dL with IVIG; use prophylaxis for *Pneumocystis jirovecii* pneumonia and herpes zoster infections; vaccinate for COVID-19; explore less frequent dosing when appropriate; and treat neutropenia with growth factor. These steps will help reduce infections, improve patient outcomes, and play a vital role in the success of anti-BCMA bispecific antibodies.

## References

[1] Moreau P, Garfall AL, van de Donk N, Nahi H, San-Miguel JF, Oriol A (2022). Teclistamab in Relapsed or Refractory Multiple Myeloma. N Engl J Med.

[2] Lesokhin AM, Tomasson MH, Arnulf B, Bahlis NJ, Miles Prince H, Niesvizky R (2023). Elranatamab in relapsed or refractory multiple myeloma: phase 2 MagnetisMM-3 trial results. Nat Med.

[3] Nooka AK, Rodriguez C, Mateos MV, Manier S, Chastain K, Banerjee A (2024). Incidence, timing, and management of infections in patients receiving teclistamab for the treatment of relapsed/refractory multiple myeloma in the MajesTEC-1 study. Cancer.

[4] Berdeja JG, Madduri D, Usmani SZ, Jakubowiak A, Agha M, Cohen AD (2021). Ciltacabtagene autoleucel, a B-cell maturation antigen-directed chimeric antigen receptor T-cell therapy in patients with relapsed or refractory multiple myeloma (CARTITUDE-1): a phase 1b/2 open-label study. Lancet.

[5] Lin Y, Martin TG, Usmani SZ, Berdeja JG, Jakubowiak AJ, Agha ME (2023). CARTITUDE-1 final results: Phase 1b/2 study of ciltacabtagene autoleucel in heavily pretreated patients with relapsed/refractory multiple myeloma. J Clin Oncol.

[6] Caro J, Braunstein M, Williams L, Bruno B, Kaminetzky D, Siegel A (2022). Inflammation and infection in plasma cell disorders: how pathogens shape the fate of patients. Leukemia.

[7] Blimark C, Holmberg E, Mellqvist UH, Landgren O, Bjorkholm M, Hultcrantz M (2015). Multiple myeloma and infections: a population-based study on 9253 multiple myeloma patients. Haematologica.

[8] O’Connor BP, Raman VS, Erickson LD, Cook WJ, Weaver LK, Ahonen C (2004). BCMA is essential for the survival of long-lived bone marrow plasma cells. J Exp Med.

[9] Cho SF, Anderson KC, Tai YT (2018). Targeting B Cell Maturation Antigen (BCMA) in Multiple Myeloma: Potential Uses of BCMA-Based Immunotherapy. Front Immunol.

[10] Frerichs KA, Verkleij CPM, Mateos MV, Martin TG, Rodriguez C, Nooka A (2024). Teclistamab impairs humoral immunity in patients with heavily pretreated myeloma: importance of immunoglobulin supplementation. Blood Adv.

[11] Smith EL, Harrington K, Staehr M, Masakayan R, Jones J, Long TJ (2019). GPRC5D is a target for the immunotherapy of multiple myeloma with rationally designed CAR T cells. Sci Transl Med.

[12] Chari A, Minnema MC, Berdeja JG, Oriol A, van de Donk N, Rodriguez-Otero P (2022). Talquetamab, a T-Cell-Redirecting GPRC5D Bispecific Antibody for Multiple Myeloma. N Engl J Med.

[13] Lancman G, Parsa K, Kotlarz K, Avery L, Lurie A, Lieberman-Cribbin A (2023). IVIg Use Associated with Ten-Fold Reduction of Serious Infections in Multiple Myeloma Patients Treated with Anti-BCMA Bispecific Antibodies. Blood Cancer Discov.

[14] Raje N, Anderson K, Einsele H, Efebera Y, Gay F, Hammond SP (2023). Monitoring, prophylaxis, and treatment of infections in patients with MM receiving bispecific antibody therapy: consensus recommendations from an expert panel. Blood Cancer J.

[15] Ludwig H, Terpos E, van de Donk N, Mateos MV, Moreau P, Dimopoulos MA (2023). Prevention and management of adverse events during treatment with bispecific antibodies and CAR T cells in multiple myeloma: a consensus report of the European Myeloma Network. Lancet Oncol.

[16] Raje NS, Anaissie E, Kumar SK, Lonial S, Martin T, Gertz MA (2022). Consensus guidelines and recommendations for infection prevention in multiple myeloma: a report from the International Myeloma Working Group. Lancet Haematol.

[17] Usmani SZ, Karlin L, Benboubker L, Nahi H, San-Miguel J, Trancucci D (2023). Durability of responses with biweekly dosing of teclistamab in patients with relapsed/refractory multiple myeloma achieving a clinical response in the majesTEC-1 study. J Clin Oncol.

[18] Razzo B, Grady C, Susanibar-Adaniya S, Waxman A, Vogl DT, Cohen AD (2023). A Phase 2, Single-Arm, Non-Inferiority Study of Limited-Duration Teclistamab for Relapsed and Refractory Multiple Myeloma (LimiTec). Blood.

[19] Philipp N, Kazerani M, Nicholls A, Vick B, Wulf J, Straub T (2022). T-cell exhaustion induced by continuous bispecific molecule exposure is ameliorated by treatment-free intervals. Blood.

[20] Dima D, Davis JA, Ahmed N, Sannareddy A, Shaikh H, Mahmoudjafari Z (2023). Real-World Safety and Efficacy of Teclistamab for Patients with Heavily Pretreated Relapsed-Refractory Multiple Myeloma. Blood.

[21] Riedhammer C, Bassermann F, Besemer B, Bewarder M, Brunner F, Carpinteiro A (2024). Real-world analysis of teclistamab in 123 RRMM patients from Germany. Leukemia.

